# Supplementary material for: The interactome of intact mitochondria by cross-linking mass spectrometry provides evidence for coexisting respiratory supercomplexes
Source: Mol Cell Proteomics. 2017 Dec 8;17(2):216–32. doi: 10.1074/mcp.RA117.000470 (PMC5795388; doi:10.1074/mcp.RA117.000470)
Supplement: Supplemental Data [file supp_RA117.000470_133922_0_supp_23978_fzffwf.zip › spectra_annotation/mito_spectra_annotation/156-1-15-1-78-1.pdf]

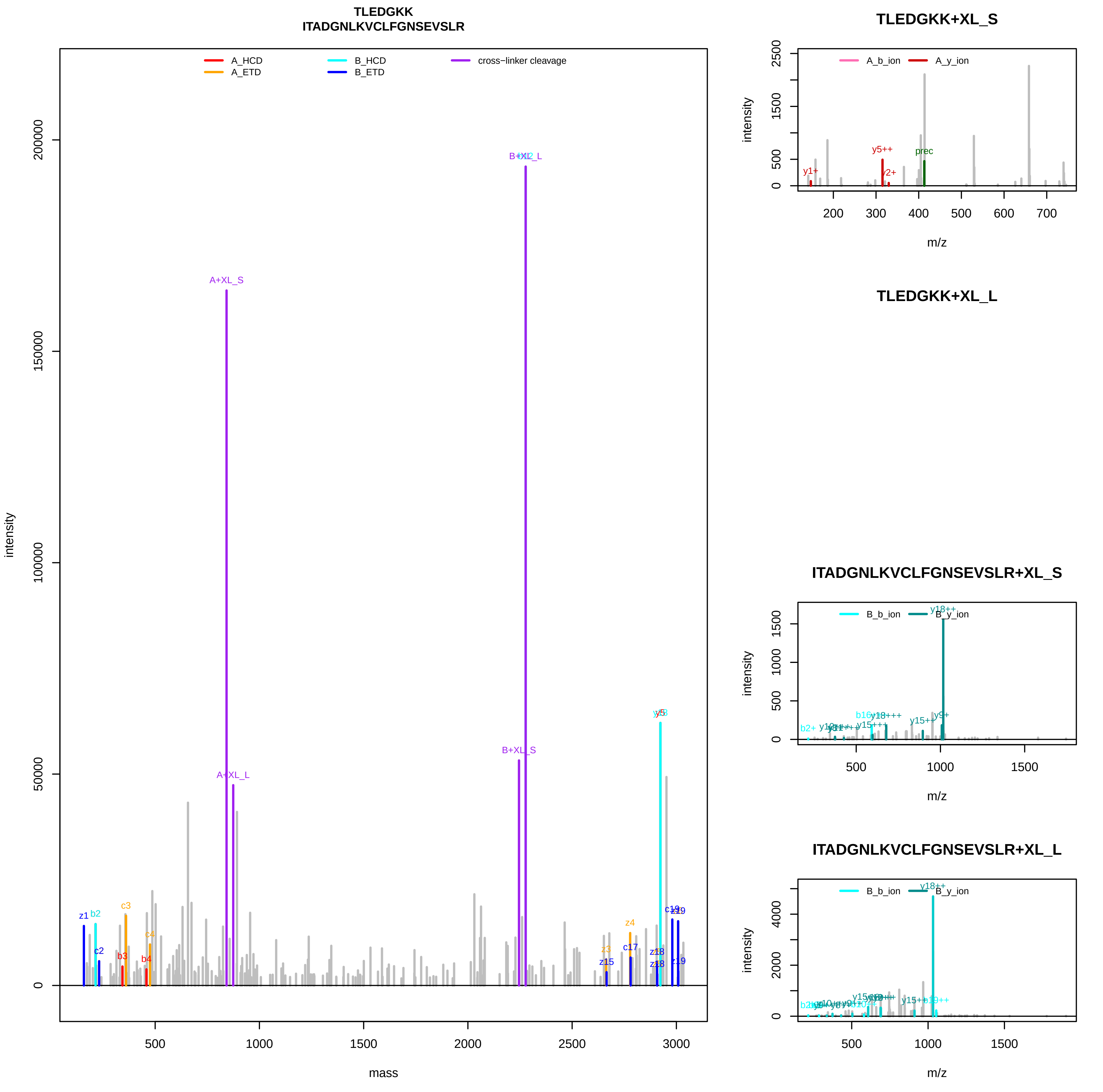
TLEDGKK
ITADGNLKVCLFGNSEVSLR
A_HCD
A_ETD
B_HCD
B_ETD
cross−linker cleavage
A+XL_S
A+XL_L
B+XL_S
B+XL_L
z1
b2
c2
b3
c3
b4
c4
z3
z4
z15
c17
z18
c19
z19
y5
intensity
mass
0
50000
100000
150000
200000
500
1000
1500
2000
2500
3000
TLEDGKK+XL_S
A_b_ion
A_y_ion
y1+
y5++
y2+
prec
m/z
TLEDGKK+XL_L
ITADGNLKVCLFGNSEVSLR+XL_S
B_b_ion
B_y_ion
y18++
b2+
b16
y18+++
y15+++
y15++
y9+
ITADGNLKVCLFGNSEVSLR+XL_L
y15++
b19++
